# Disparity in reimbursement for tuberculosis care among different health insurance schemes: evidence from three counties in central China

**DOI:** 10.1186/s40249-016-0102-4

**Published:** 2016-01-27

**Authors:** Yao Pan, Shanquan Chen, Manli Chen, Pei Zhang, Qian Long, Li Xiang, Henry Lucas

**Affiliations:** School of Medicine and Health Management, Huazhong University of Science and Technology, Wuhan, China; The Third Affiliated Hospital, Sun Yat-sen University, Guangzhou, China; The Jockey Club School of Public Health and Primary Care, The Chinese University of Hong Kong, Hong Kong, China; School of Management, Hubei University of Chinese Medicine, Wuhan, China; Yichang Center for Disease Control and Prevention, Yichang, China; Duke Global Health Institute, Duke University, Durham, NC USA; Global Health Research Center, Duke Kunshan University, Kunshan, China; Institute of Development Studies, Sussex University, Brighton, UK

**Keywords:** Disparity, Tuberculosis care, Health insurance schemes, Equity, China

## Abstract

**Background:**

Health inequity is an important issue all around the world. The Chinese basic medical security system comprises three major insurance schemes, namely the Urban Employee Basic Medical Insurance (UEBMI), the Urban Resident Basic Medical Insurance (URBMI), and the New Cooperative Medical Scheme (NCMS). Little research has been conducted to look into the disparity in payments among the health insurance schemes in China. In this study, we aimed to evaluate the disparity in reimbursements for tuberculosis (TB) care among the abovementioned health insurance schemes.

**Methods:**

This study uses a World Health Organization (WHO) framework to analyze the disparities and equity relating to the three dimensions of health insurance: population coverage, the range of services covered, and the extent to which costs are covered. Each of the health insurance scheme’s policies were categorized and analyzed. An analysis of the claims database of all hospitalizations reimbursed from 2010 to 2012 in three counties of Yichang city (YC), which included 1506 discharges, was conducted to identify the differences in reimbursement rates and out-of-pocket (OOP) expenses among the health insurance schemes.

**Results:**

Tuberculosis patients had various inpatient expenses depending on which scheme they were covered by (TB patients covered by the NCMS have less inpatient expenses than those who were covered by the URBMI, who have less inpatient expenses than those covered by the UEBMI). We found a significant horizontal inequity of healthcare utilization among the lower socioeconomic groups. In terms of financial inequity, TB patients who earned less paid more. The NCMS provides modest financial protection, based on income. Overall, TB patients from lower socioeconomic groups were the most vulnerable.

**Conclusion:**

There are large disparities in reimbursement for TB care among the three health insurance schemes and this, in turn, hampers TB control. Reducing the gap in health outcomes between the three health insurance schemes in China should be a focus of TB care and control. Achieving equity through integrated policies that avoid discrimination is likely to be effective.

**Electronic supplementary material:**

The online version of this article (doi:10.1186/s40249-016-0102-4) contains supplementary material, which is available to authorized users.

## Multilingual abstract

Please see Additional file 1 for translation of the abstract into the six official working languages of the United Nations.

## Background

China’s health care system is bifurcated in nature between rural and urban areas [1, 2]. There are three major government-led complementary insurance schemes collectively known as the Chinese basic medical security system. The schemes are the Urban Employee Basic Medical Insurance (UEBMI), the Urban Resident Basic Medical Insurance (URBMI), and the New Cooperative Medical Scheme (NCMS) [3, 4]. The UEBMI-established in 1998 and designed exclusively for urban workers, including both public and private sector employees, and retirees-is a mandatory scheme based on cost sharing between employers and employees, with risk pooling managed at the city level [5]. The URBMI, which was introduced in 2007, is for urban residents without formal employment who are not covered by the UEBMI (e.g., students, young children, the elderly, disabled and other unemployed urban residents), and is co-financed by those who use it and the local government. It is managed at multiple levels. The NCMS is a voluntary program designed to deal with catastrophic illnesses at the county level. It is based on cost sharing between the government and farmers, and aims to improve access to health insurance for the rural population [6, 7]. Counties determine benefit packages and administrative arrangements according to their local conditions. The NCMS was piloted in 2003 and has been expanded to 2566 participating counties, covering 98.3 % of the target population, in 2012.

The source and level of financing for the three health insurance schemes are different, thus resulting in different reimbursement levels and anti-risk capacity. For the UEBMI, the annual premium is made up of 8 % of employees’ wages, among which 6 % is contributed by employers’ payroll tax, and 2 % is contributed by the employees themselves. For the URBMI, the annual premium in 2008 was on average 245 RMB for adults and 113 RMB for minors. In 2012, the annual NCMS premium was 300 RMB (made up of 240 RMB from central and local governments, and 60 RMB from individuals).

Each scheme provides different levels of reimbursement [8]. Better access to healthcare and risk protection have been achieved through the expansion of insurance coverage (reaching 95.7 % in 2011 [9]), and increase in subsidies and benefits over time. Prior studies that have evaluated disparities in health insurance generally focused on one scheme [10–13], and little is known about the disparities among the different health insurance schemes. Several studies have reported that families covered by the UEBMI or the URBMI had lower rates of catastrophic health expenditure than those enrolled in the NCMS, however, these studies were neither systematic nor specific [14]. The levels of reimbursement and associated out-of-pocket (OOP) expenses related to a given disease among the different health insurance schemes have not been studied. The primary objective of this study is to fill this evidence gap by examining disparities in reimbursement for tuberculosis (TB) care among the health insurance schemes in China.

Because TB is an infectious disease, it is ideal for this study. China has the second largest TB burden in the world [15] and the disease has long been on the governmental agenda [16]. Considerable progress has been made towards addressing the TB epidemic, however, TB treatment costs remain a heavy financial burden on patients [17, 18]. In China, treatment for TB is free in theory, but studies show that there are many associated healthcare costs, such as liver-protection drugs and extra diagnostic tests, as well as considerable indirect costs [19–21].

As a key policy area for TB care and prevention [22, 23], health insurance is an indispensable means of financial protection [24]. Integrating the national TB control program into health insurance schemes is an effective strategy to address challenges in current China [25]. In China, TB patients can receive anti-TB treatment in designated hospitals through the coverage of the three health insurance schemes. Expenses associated with TB treatment may be partially covered by these schemes, however, patients are responsible for any required deductibles and co-payments. An evaluation of the disparity in reimbursements for TB care among the health insurance schemes would inform how to best design the reimbursement structure to ensure both equity and efficiency in TB control.

This study extended previous studies by analyzing reimbursement related to TB care among the abovementioned health insurance schemes. A claims database analysis of all hospitalization reimbursed by the schemes in Yichang city (YC), central China, covering 1506 discharges, was conducted to identify differences in the total inpatient expenses, OOP expenses and the effective reimbursement rate.

## Methods

### Study setting

According to a study published in *The Lancet*, the lowest average inpatient reimbursement rates were reported in China’s central region (41.2 %). Households in the central region are vulnerable, with high rates of catastrophic health spending. The percentage of households experiencing catastrophic health expenses was 13.7 % (13.3 % in the west region and 11.9 % in the east region). In 2011, households in the central region spent an average of 13.2 % of their annual expenditure on health (13.1 % in the west region and 12.4 % in the east region) [9]. For this reason, this study focuses on the central region.

Located in central China and the middle reaches of Yangtze River, the Hubei province had a gross domestic product (GDP) per capita totaling RMB 34,131 in 2011, which ranks it 13th among the 32 provinces (municipalities and autonomous regions) in China’s mainland. Yichang city, located in southwestern Hubei, had a GDP per capita of RMB 56,265 in 2011. The China National Health and Family Planning Commission (NHFPC)-Bill & Melinda Gates Foundation TB Project has been widely conducted in YC. The local governments were able and willing to collaborate in this study. Given what mentioned above, our study was designed to examine the disparity of tuberculosis care reimbursement among different health insurance schemes in YC, Hubei province.

A stratified random sampling procedure was used. Three counties in YC were purposively selected to represent the entire city, in terms of socioeconomic development and geographic conditions (hilly/plain): Yidu (YD), Zhijiang (ZJ) and Wufeng (WF) were chosen as the study counties. Yidu is the most developed county, whereas WF is the most underdeveloped one, as shown in Table 1.

**Table 1:** Economic status in three counties in YC 2012

	YD	ZJ	WF
GDP per capita (yuan)	89001	58630	23325
Urban annual income per capita (yuan)	19431	17465	12064
Rural annual income per capita (yuan)	10415	10496	4391

*Data source:* The economic indicators were obtained from statistical yearbook in YC

### Data sources

Quantitative data were obtained primarily from the routine data systems of the UEBMI, the URBMI and the NCMS, from their corresponding offices in each county. To estimate the direct medical costs of and financial burden on general TB patients in all study counties, information managers extracted reimbursement data of inpatients diagnosed with TB from January 2010 to December 2012. Key variables included sex, age group and choice of health providers, and hospitalization cost, reimbursement expenses, non-reimbursable expenses relating to TB associated service provision. 1506 discharges were conducted, including 1001 discharges of the NCMS, 348 of the UEBMI and 157 of the URBMI.

All study counties were required to collect policy documents related to payment and reimbursements of TB treatment costs.

### Conceptual framework

Equity is widely considered a major objective of healthcare policies in international settings [26]. Generally, it can be divided into three parts: health equity, financial equity and utilization equity. Equity in health means providing all population groups an equal opportunity to be healthy [27, 28]. Financial equity plays a significant role in promoting healthcare access and achieving universal coverage of health services, especially for the poor and vulnerable groups [29]. It requires that healthcare payments are determined fairly and based on a household’s ability to pay (ATP). The equity of utilization on the other hand is judged using the concentration index, as an individual’s need for health care isn’t reliant on income [30]. Financial equity and utilization equity can be defined in two dimensions: horizontal equity and vertical equity. Horizontal equity means that equal access to health care should be provided to people with the same illness (equal treatment for equal needs) [31]. Vertical equity means that people with the greatest needs are given the most care [26]. The pursuit of equity is a primary objective of healthcare systems, and health insurance is frequently cited as a key determinant of ensuring equity as it lowers financial barriers and increases demand for health care.

The World Health Report 2010 represented the concept of universal health coverage (UHC) in three dimensions: breadth, depth and height. Breadth refers to population coverage, depth refers to the range of services covered, and height refers to the extent to which costs associated with health care are covered [14, 32]. This study will use this framework to look into the disparities in reimbursement for TB care among the three health insurance schemes in China.

### Data analysis

The NCMS operates at the county level. The UEBMI and the URBMI are managed at multiple levels. Thus, we made a comparison at the county level.

The quantitative data analysis was performed using SPSS Statistics version 17.0. The main analysis used descriptive statistics and focused on the associations between health insurance, and the expenditure and reimbursement rates associated with TB-related services. To measure the level of reimbursement related to TB care, a number of things were considered, including OOP expenses, the effective reimbursement rate (amount of reimbursement/total expenditure on medical care), and the non-reimbursable expenses rate (non-reimbursable expenses/total expenditure on medical care). An analysis of the financial burden placed on patients was also conducted by dividing the OOP expenses by the annual average income per capita. Equity of access to TB care among the three health insurance schemes was assessed, disaggregated by project sites. Appropriate statistical methods, including variance analysis, were employed for the data analysis.

Health insurance policies were categorized and analyzed by region. The deductible, the reimbursement rate and the ceiling level of multi-level hospitals were also determined.

### Quality assurance

Measures were taken to ensure the quality of the data compiled. All the data collection instruments, tools and procedures developed for the study were tested in a pilot exercise in one project county. Following this exercise, a workshop was held to discuss any problems and to identify what needed to be amended. A logic check of all collected data was also done to identify gaps, inaccuracies and apparent incongruities and inconsistencies.

### Missing data

Comprehensive data from the three health insurance schemes for three years were included in the analysis, with one exception from ZJ, where only data from NCMS for the year 2012 were included in the analysis.

### Ethical approval

Ethical approval was obtained from the Institutional Ethics Committee, Chinese Center for Disease Control and Prevention, China.

## Results

### Disparities in reimbursement for TB care among the three health insurance schemes

#### Population: who is covered?

The three health insurance schemes have coverage of over 95 % of the total population in all three counties, as shown in Table 2. All three schemes offer inpatient and outpatient reimbursement for TB care in different forms.

**Table 2:** Health insurance coverage in three counties in YC 2012

	YD	ZJ	WF
NCMS coverage rate (%)	100	99	98
UEBMI coverage rate (%)	98	98	100
URBMI coverage rate (%)	98	99	96

*Data source:* Data were collected from the records of NCMS, UEBMI and UEBMI offices

The NCMS emphasizes coverage of TB inpatient services [33] and hospitalization expenditures can be reimbursed with some co-payment. There are three modes of outpatient reimbursement as part of the NCMS [34, 35]: (1) household savings accounts, which can be used by beneficiaries directly to pay for outpatient expenditures; (2) outpatient reimbursement, which reimburses outpatient fees up to a certain amount at county and/or township level; and (3) outpatient reimbursement for selected catastrophic or chronic illnesses, which compensates for large outpatient expenditures by establishing a catastrophic or chronic illness pooling fund. This includes diseases that are expensive to treat, but don’t necessarily require admission to hospital (e.g. nephropathy, hepatitis, diabetes, hypertension). All three counties adopt this three-level structure. Reimbursements for TB outpatient care are available at township, village or community health facilities. Tuberculosis patients who have to seek treatment in the county-level TB designated hospitals cannot claim reimbursement for general outpatient care. Because of this, the NCMS provides a package covering chronic diseases, including TB, which means that TB patients can claim reimbursement for outpatient care accordingly (see Table 3).

**Table 3:** Reimbursement policies of three health insurance schemes for TB outpatient services

Health insurance scheme	Inpatient reimbursement	Outpatient reimbursement	
		General outpatient	Chronic diseases outpatient
NCMS	√	×	√
UEBMI	√	×	√
URBMI	√	√	×

*Note:* “√” indicates TB patients can enjoy the compensation policy and vice versa

The UEBMI consists of a pooled fund for inpatient stays and individual medical savings accounts for outpatient visits [36]. In terms of TB care, the UEBMI offers inpatient and outpatient reimbursement for chronic diseases (a similar structure to the NCMS).

The URBMI seeks to eliminate impoverishment caused by high medical expenses by focusing on inpatient and outpatient services for chronic and fatal diseases, such as diabetes and heart disease [37]. Tuberculosis patients are not covered by a package covering services for chronic diseases, however, they can still claim for inpatient and general outpatient reimbursement.

#### Services: which services are covered?

The NCMS offers a narrower benefit package than the other two schemes. Eleven anti-TB drugs are included in the NCMS, namely streptomycin, isoniazid, rifampicin, ethambutol, aminosalicylate sodium, pyrazinamide, rifapentine and rifamycin, among others, whereas 20 drugs are covered by the UEBMI and the URBMI. Drugs are estimated to account for just under half of a TB patient’s OOP expenses [38]. A more inclusive drug reimbursement list could help reduce TB patients’ OOP expenses by decreasing non-reimbursable expenses. According to our data results related to TB inpatients, non-reimbursable expenses rates for those covered by the NCMS from 2010 to 2012 in YD, ZJ and WF were 7.8, 13.34 and 5.8 %, respectively.

#### Costs: proportion of the costs covered

The ability of health insurance schemes to reduce the financial burden of patients depends on the amount of funds that can be raised and pooled. Compared to the UEBMI, the URBMI and the NCMS have low financing. In YC city, for the NCMS, the annual premium per person was RMB 290 in 2012; for the URBMI, it was RMB 200; and for the UEBMI, it was usually over RMB 1000. Thus, the NCMS and URBMI have very basic benefit packages, which means they don’t provide their beneficiaries with adequate funds to alleviate the economic hardships caused by serious diseases. Table 4 shows the reimbursement rates for TB outpatient care among the different health insurance schemes, by area.

**Table 4:** Reimbursement of three health insurance schemes for TB outpatient care in 2012

Health insurance scheme	City/County	Outpatient reimbursement
NCMS	YD	200 yuan/year
	ZJ	45 yuan/month with an annual reimbursement cap of 540 yuan
	WF	70 % of annual total costwith an annual reimbursement cap of 2000 yuan
UEBMI	YC	reimbursement rate averages 75 % within 200 yuan/month (6 months for first treatment, 8 months for retreatment)
URBMI	YC	40 % of accumulated cost ranging from 50 to 400 yuan

Tuberculosis patients who are covered by the UEBMI enjoy a more generous inpatient reimbursement policy, as shown in Table 5. In this scheme, overall reimbursement rates increase with medical expenses rather than being determined by the level of the medical institution/hospital where the patient sought treatment. The ceiling is four times that of the average wage in the locality.

**Table 5:** Deductible (in Yuan) and reimbursement rates (percentages) by hospital type and health insurance type in YC

County	Health insurance scheme	Deductible (Yuan)			Reimbursement rate (%)		
		THC	CH	CHHL	THC	CH	CHHL
YD	NCMS	100	500	500	85	65	50–65
	UEBMI	—	—	—	85–95	85–95	85–95
	URBMI	100	300	500	80	60	50
ZJ	NCMS	100	300	300–500	85	75	50–65
	UEBMI	—	—	—	85–95	85–95	85–95
	URBMI	100	300	500	80	60	50
WF	NCMS	50	200	500	80	70	55–65
	UEBMI	—	—	—	85–95	85–95	85–95
	URBMI	100	300	500	80	60	50

*Note:* Reimbursement rate of UEBMI varies with medical cost section instead of hospital level

THC refers to “township health centers”

CH refers to “county hospitals”

“CHHL” refers to “city hospitals or higher level”

*Data resource:* Author’s data are collected from the records of NCMS, UEBMI and UEBMI offices

The NCMS reimbursement rates are higher than those of the URBMI, but lower than those of the UEBMI. Reimbursement for TB inpatient services is the same as for other inpatient services covered by the NCMS. The higher the level of the medical institution in which a patient receives treatment, the more he/she needs to pay out of pocket. The ceiling level for reimbursement ranged from RMB 100,000 to RMB 150,000.

### The impact of disparities in reimbursement for TB care among the different health insurance schemes

Tuberculosis patients have inadequate outpatient service coverage because of limited funding, as shown in Table 4. Databases from the health insurance schemes cannot fully reflect the reimbursement rates of TB patients in the outpatient setting. Problems in the design of the information systems result in a lack of essential information about outpatient services. Therefore, we focused on examining disparities in reimbursement for TB care in the inpatient setting.

**Fig. 1:**
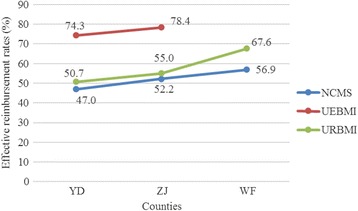
Effective reimbursement rates for TB inpatients in the three study counties in 2012 (%)

Given the different economic development levels of the counties and reimbursement levels of the schemes, we analyzed the total inpatient expenses and OOP expenses of patients enrolled in the three health insurance schemes (see Table 6). Generally, total inpatient expenses for those covered by the UEBMI were the highest. Inpatients covered by the URBMI had the highest OOP expenses. The total inpatient expenses among the three health insurance schemes can be illustrated as such: NCMS < URBMI < UEBMI, with one exception in ZJ, where the total inpatient expenses of patients covered by the NCMS were a little higher than of those patients covered by the URBMI. The OOP expenses among the three health insurance schemes can be illustrated as such: UEBMI < NCMS < URBMI, with an exception in ZJ, where it was URBMI < NCMS. Finally, the effective reimbursement rate among the three health insurance schemes can be illustrated as such: UEBMI > URBMI > NCMS (see Fig. 1).

**Table 6:** Total inpatient expense and OOP of TB inpatients in three counties in YC 2012

		Total inpatient expenses (Yuan)		OOP (Yuan)	
		Mean	Median	Mean	Median
YD	NCMS	4522	3106	2397	1513
	UEBMI	7371	4197	1895	1097
	URBMI	5525	2964	2722	1489
ZJ	NCMS	6372	4734	3048	1972
	UEBMI	7583	4376	1634	1027
	URBMI	6333	4190	2725	1729
WF	NCMS	4818	3148	2078	1195
	URBMI	11491	6443	3495	2121

*Note:* WF is a typical agricultural county with a smaller urban population, and there were no TB patients with UEBMI

**Fig. 2:**
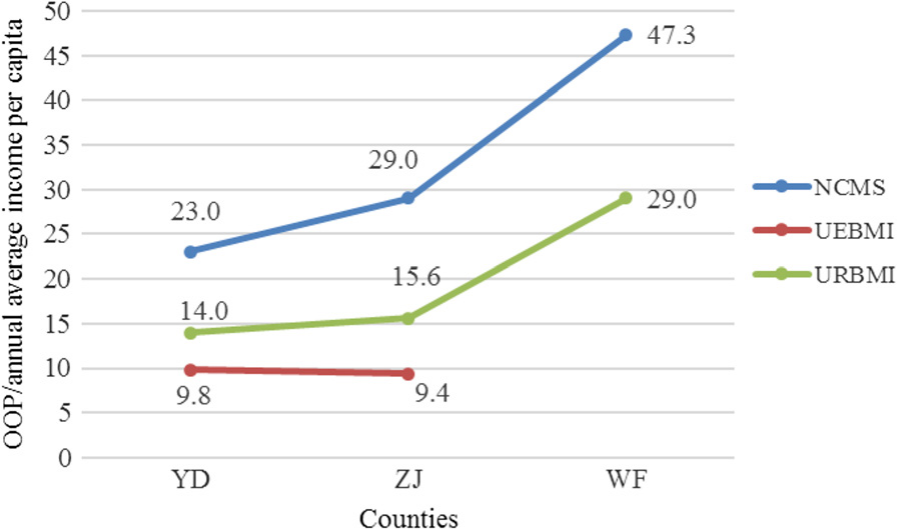
Numbers calculated by dividing the OOP expenses by the annual average income per capita in YC, by health insurance type

We further analyzed patients’ choice of health providers by health insurance scheme (see Table 7). Tuberculosis patients covered by the UEBMI could get almost equivalent reimbursement rates in all medical institutions. The OOP expenses of patients covered by the URBMI and NCMS generally increased at higher levels of the referral system, whereas reimbursement rates decreased according to the policy design. We can conclude that the design of the reimbursement policy had a significant impact on patients’ medical-seeking behavior.

**Table 7:** Number of discharges, mean OOP and effective reimbursement rate per hospitalization by health insurance scheme type in YC City

	Number (%)			Mean OOP per hospitalization (Yuan)			Effective reimbursement rate (%)		
	Township	County	Prefecture/Province	Township	County	Prefecture/Province	Township	County	Prefecture/Province
NCMS	161 (16.1)	515 (51.5)	325 (32.5)	355	1708	4655	75.1	57.7	45.2
UEBMI	9 (2.6)	237 (68.1)	102 (29.3)	554	1192	3177	76.3	75.5	77.3
URBMI	5 (3.3)	112 (73.2)	36 (23.5)	350	1802	5921	70.6	57.0	49.6

*Note:* The differences in mean OOP and effective reimbursement rate per hospitalization by health insurance scheme type were all significant at *P* < 0.05 according to analysis of variance

To further estimate the financial burden placed on TB patients covered by the different health insurance schemes, we divided the OOP expenses by the annual average income per capita. Shows that NCMS provided modest financial protection, based on income. Generally, the number calculated by dividing “OOP expenses by the annual average income per capita” was higher in poorer counties Fig. 2.

## Discussion

To the best of our knowledge, limited research has been conducted on the disparities in health insurance schemes. Health disparities are a serious public health issue in the USA [39], where insurance systems are decentralized. Several studies have focused on insurance-related disparities to determine whether there are differences in treatment based on the insurance patients are covered by. By examining a single medical condition, several studies have found that insurance status was associated with different mortality outcomes and use of resources [40–43]. Hasan et al. reported that patients covered by Medicare or private insurance tend to receive higher-quality care than those covered by Medicaid [44]. In a comparison of quality of care delivered to patients in the same hospital, Spencer et al. found that there are differences in quality of health care provided to patients across different payer types even within the same hospital [45]. Rashford et al. also found that a single-payer system would cost less to manage than a multi-payer system [46]. Using data from the Fourth National Health Service Survey, one study published in Chinese found that the populations covered by the UEBMI had a higher benefit level and lower economic burden than those covered by the URBMI or NCMS [47]. The present study is the first in China to compare the disparities in reimbursements for a given disease among different health insurance schemes. Therefore, this study can control the biases associated with various types of illness and obtain more accurate results. Three key findings emerge from this analysis, which we discuss below.

Although TB patients covered by the three health insurance schemes suffer from the same illness, our study showed that they have different inpatient expenses (TB patients covered by the NCMS have less inpatient expenses than those who were covered by the URBMI, who have less inpatient expenses than those covered by the UEBMI). This indicates a significant horizontal inequity of healthcare utilization in the lower socioeconomic groups. Those covered by the UEBMI had almost equivalent reimbursement rates in all medical institutions, thus they could seek medical care at higher level medical institutions, which helps to ensure a relatively high quality of medical care. Reimbursement levels for patients covered by the URBMI and NCMS varied by hospital type. In particular, TB patients covered by the NCMS were disadvantaged in accessing TB inpatient care, which is consistent with previous reports showing how different types of health insurance programs affect healthcare utilization outcomes among the elderly in China [48]. There could be two reasons for this. First, TB patients covered by the NCMS are usually from the lower socioeconomic bracket. Second, TB patients covered by the NCMS were located in rural areas and their access to health care was usually hindered by poor transportation services. Low incomes and inadequate reimbursement rates also led to curtailed access. Tuberculosis patients may not seek medical treatment because of financial issues or incur catastrophic health expenses if they continue treatment, both of which would hamper TB control [49].

Our study also revealed financial inequity among TB patients. The prepayment structure of health insurance schemes is supposed to shift funds from the rich to the poor. But according to our results, TB patients who earned less actually paid more.

Generally, TB patients are more likely to receive outpatient treatment rather than seek inpatient services. The NCMS and URBMI mainly cover reimbursement for hospitalization, and their reimbursement structures for TB outpatient services are not adequate, characterized by low ceilings and high co-insurance rates. The UEBMI offered a more generous outpatient reimbursement policy with a higher reimbursement rate and an annual reimbursement cap. But it is still not sufficient. Existing literature shows that low-income patients who incur high costs for TB treatment have a poor adherence for treatment, which leads to interrupted or suspended treatment [50]. Given the inadequate outpatient reimbursement rates and the length of outpatient treatment, a portion of poor TB patients may fail to complete their treatment. Meanwhile, TB patients who have higher incomes may seek inpatient treatment that is reimbursed at a higher rate, which results in a large number of hospitalizations and places a severe economic burden on patients.

The UEBMI provides comprehensive services for insured TB inpatients, with higher reimbursement ratios. Hence, TB patients covered by the UEBMI had the highest effective reimbursement rate (exceeding 75 %) and the lowest mean OOP expenses (less than RMB 2000). The URBMI and the NCMS provide neither adequate financial protection nor service coverage for TB patients. Taking income into account, TB patients covered by the NCMS were particularly vulnerable; their expense rates indicated a high risk for catastrophic health spending. A large proportion of the inpatient expenses had to be covered by the individual or his/her family, which impairs affordable and equitable access to health care and consequently hampers TB control. Furthermore, TB patients covered by the NCMS usually have low ATP. The number calculated by dividing OOP expenses by the annual average income per capita was significantly higher among NCMS beneficiaries than among the UEBMI beneficiaries who have higher ATP and it was not progressive to ATP, suggesting that the worse off TB patients were required to pay more out of pocket.

There are two alternative explanations for the financial inequity among the different health insurance schemes. First, the relatively low financing level results in a comparatively low level of compensation for medical expenses [33, 51]. Second, the three health insurance schemes cover specific groups: rural residents (under the NCMS), urban employees (under the UEBMI), and unemployed urban residents (under the URBMI). Generally, TB inpatients who are covered by the URBMI or the NCMS are more vulnerable and have lower ATP. They also often have poor healthcare consciousness and access.

Prior studies have showed that a patient’s economic status plays an important role in financial burden alleviation [52, 53]. Our study also highlighted that TB patients in poorer areas were more vulnerable. The number calculated by dividing OOP expenses by the annual average income per capita decreased progressively with the economic level of the studied counties. This relates to findings from other studies, which found that poverty is both a cause and a devastating outcome of TB [54]. Medical financial assistance for the poor has been established in almost all regions of China. However, the criteria to be eligible for this assistance are often very demanding and most TB patients are not qualified to apply, unless specific policies have been developed to meet their needs.

This study had several limitations, hence, the conclusions drawn from this paper should be applied with caution. First, the sample size is relatively small. Only samples from one city in central China were considered. Second, previous studies concerning inequity are all based on income groups of the study participants. Due to the limitation of the information system, we couldn’t gather information about the income of every TB patient in this study. Instead, we made estimates using average incomes, which may narrow the differences of income-related equity among TB patients.

## Conclusion

Our study had three major findings. First, there is a significant horizontal inequity of healthcare utilization among TB patients from lower socioeconomic groups. Second, poorer TB patients who are covered by the NCMS paid more out of pocket. Third, TB patients in the lower socioeconomic bracket were more vulnerable.

These findings have important policy implications, especially in the context of TB control, implementation of health insurance schemes, and broader reforms of health equity. Further priorities could focus on improving healthcare equities in outcomes, controlling TB inpatient costs and addressing major concerns about the benefit packages of health insurance schemes.

First, an important implication is that the disparity in reimbursement for TB care among the different health insurance schemes would eventually hamper TB control. Reducing the gap in health outcomes, i.e. reimbursement rates, among the three health insurance schemes in China should be a focus of TB care and control. Tuberculosis patients covered by the URBMI or the NCMS should be the target population of future healthcare policies on TB.

This implication has an international significance. First, our study provides a solution for how health insurance schemes can be effective in TB control. According to the WHO, in 2013, nine million people fell ill with TB and 1.5 million died from the disease. Strategies are urgently needed to end the global TB epidemic. Second, UHC is an increasingly important global initiative. Our study of the China experience showed large disparities in reimbursement for TB care among the three health insurance schemes in the three dimensions of UHC, uncovering additional evidence about the impact of different health insurance benefit packages on TB health service utilization. In addition, the distributional results of health insurance expansion efforts could be enlightening, especially for countries with decentralized insurance systems. In order to achieve UHC, measures should be taken to improve health outcomes and tackle poverty by narrowing gaps and increasing the coverage of different health insurance schemes.

Second, inequity is a common challenge worldwide. The present study reveals that there is inequity within counties, which may provide useful lessons for countries with decentralized insurance systems that face similar challenges. In addition, this study indicates that inequity could be reduced by tailoring health insurance policies. As for China, given the differences in reimbursement rates among the UEBMI, the URBMI and the NCMS, achieving equity through integrated policies that avoid discrimination is likely to be effective.

## Additional file

Additional file 1:
**Multilingual abstracts in the six official working languages of the United Nations.** (PDF 267 kb)

## Abbreviations

ATP: Ability-to-pay
NCMS: New Cooperative Medical Scheme
OOP: Out-of-pocket
TB: tuberculosis
UEBMI: Urban Employee Basic Medical Insurance
UHC: Universal health coverage
URBMI: Urban Resident Basic Medical Insurance
WF: Wufeng county
WHO: World Health Organization
YC: Yichang City
YD: Yidu county
ZJ: Zhijiang county
