# Supplementary material for: Disparity in reimbursement for tuberculosis care among different health insurance schemes: evidence from three counties in central China
Source: Infect Dis Poverty. 2016 Jan 27;5:7. doi: 10.1186/s40249-016-0102-4 (PMC4729161; doi:10.1186/s40249-016-0102-4)
Supplement: Additional file 1: — Multilingual abstracts in the six official working languages of the United Nations. (PDF 267 kb) [file 40249_2016_102_MOESM1_ESM.pdf]

Translation of the abstract into the six official working languages of the United Nations

التفاوت في صرف قيمة تكاليف رعاية مرضى السل بين برامج التأمين الصحي المختلفة: أدلة من ثلاث مقاطعات في وسط الصين

ياو بان، شانكوان تشن، مانلي تشن، بي تزانج، تشيان لونغ، لي زيانج، هنري لوكاس

الملخص:

الخلفية: ظاهرة عدم العدالة في مجال الرعاية الصحية يعتبر مسألة مهمة في جميع أنحاء العالم. يتكون نظام التأمين الطبي الأساسي في الصين من ثلاثة برامج تأمين رئيسية، و هي: برنامج التأمين الطبي الأساسي للموظفين ؛ و برنامج التأمين الصحي لسكان المدن؛ و و برنامج التعاون الطبي الجديد. و هناك القليل من البحوث التي أجريب للبحث في مسألة عدم العدالة في عمليات الصرف بين برامج التأمين الصحي في الصين. و في هذه الدراسة، نهدف إلى تقييم التفاوت في مبالغ الصرف المدفوعة لرعاية مرضى السل (الدرن) من قبل برامج التأمين الصحي المشار إليها أعلاه.

منهجية البحث :تستخدم هذه الدراسةالإطارالخاص بمنظمة الصحة العالمية (WHO) لأغراض تحليل حالات التفاوت و العدالة فيما يتعلق بالأبعاد الثلاثة للتأمين الصحي: التغطية السكانية، ونطاق الخدمات التي تغطيها، ومدى تغطية التكاليف. تم تصنيف و تحليل السياسات الخاصة بكل من نظم التأمين الصحي، و كذلك تحليل قاعدة بيانات المطالبات لكافة الحالات التي أدخلت للعلاج في المستشفيات و تم صرف قيمتها للفترة من 2010إلى 2012 في ثلاث مقاطعات تابعة لمدينة ييتشانغ و التي تضمنت عدد 1506 من الحالات التي خرجت من المستشفى حيث جرت الدراسة لتحديد الفروقات بين معدلات المبالغ المسددة و المصروفات (خارج الجيب) بين برامج التأمين الصحي.

النتائج: تحمل مرضى السل مصروفات متعددة تبعاً لبرنامج التأمين المشمولين ضمنه، و لوحظ أن مرضى السل ضمن برنامج التأمين NCMS كانت مصروفاتهم في العيادات الداخلية أقل من هؤلاء المشمولين ببرنامج التأمين URBMI الذين انخفضت نفقاتهم في العيادات الداخلية عن المرضى التابعين لبرنامج التأمين الصحي UEBMI. و قد وجدنا معدلات كبيرة افقية من عدم العدالة بين الفئات منخفضة الأوضاع اجتماعيا واقتصاديا؛ و من ناحية عدم العدالة المالية ، فقد كان مرضى السل منخفضي الدخل يدفعون أعلى من الفئات الأخرى. يوفر برنامج NCMS حماية مالية متواضعة على أساس معدل الدخل . عموما، فإن مرضى السل من أدنى الفئات منخفضة الأوضاع اجتماعيا-اقتصاديا هي الأكثر تضرراً.

الخلاصة :هناك تفاوت ملحوظ في عمليات سداد تكاليف الرعاية الصحية لمرضى السل بين ثلاثة برامج للتأمين الصحي، وهذا بدوره، يعوق جهود مكافحة مرض السل. و ينبغي تركيز الجهود على خفض الفجوة في مخرجات الرعاية الصحية بين برامج التأمين الصحي الثلاث في الصين، و .كما أن تحقيق العدالة من خلال تطبيق سياسات متكاملة تعمل على تجنب التمييز من شأنه تحقيق نتائج إيجابية.

Translated from English version into Arabic by Lamya, through

TRANSLATORS
WITHOUT BORDERS

不同医疗保障制度下结核病患者的补偿差异性——基于中国中部地区三个县的研究

潘瑶，陈山泉，陈曼莉，张培，龙倩，项莉，Henry Lucas

摘要：

引言：健康不公平是世界的一项重要议题。中国基本医疗保障制度主要包括三种制度：城镇职工基本医疗保险（以下简称“城职”）、城镇居民基本医疗保险（以下简称“城居”）和新型农村合作医疗（以下简称“新农合”）。目前，针对中国三种不同保障制度之间差异性的研究还比较少。本研究试图研究三种不同保障制度对结核病患者补偿的差异性。

方法：本研究运用了世界卫生组织提出的理念框架，从三个维度来分析保障制度之间的差异性和公平性：人口、服务和直接

费用。各个保障制度的政策都进行了归类分析。宜昌市三个县 2010 至 2012 年的补偿数据库被用于分析不同保障制度在补偿比和自付费用上的差异。一共有 1506 个患者被纳入分析。
结果：结核病患者参与的保障制度不同，其住院费用也不同（新农合结核病患者的住院费用最低，其次为城居结核病患者，城职结核病患者住院费用最高）。研究发现不同经济水平患者之间存在卫生服务利用的不公平性。从筹资公平的角度而言，支付能力低下的结核病患者需要支付更为高额的医疗费用。同时，新农合提供的风险保护最为有限。社会经济地位低下的结核病患者是最为弱势的群体。
结论：三大保障制度在结核病的补偿上存在较大的差异。这将不利于结核病防控。缩小不同保障制度之间的差异性，是结核病防控的重点领域。通过政策的整合来保障公平，消除差异，是行之有效的方法。

Translated from English version into Chinese by Xiang Li

Disparité de remboursement des soins de prise en charge de la tuberculose parmi différents régimes d'assurance maladie : des données probantes provenant de trois départements de Chine centrale

Yao Pan, Shanquan Chen, Manli Chen, Pei Zhang, Qian Long, Li Xiang, Henry Lucas

Résumé

Contexte: Les inégalités en matière de santé constituent un important problème dans le monde entier. Le système d'assurance médicale de base chinois comprend trois principaux régimes d'assurance maladie : l'Assurance médicale de base pour les employés urbains (UEBMI), l'Assurance médicale de base pour les résidents urbains (URBMI) et le Nouveau régime médical coopératif (NCMS). Peu de recherches ont été menées afin d'analyser la disparité des paiements parmi les régimes d'assurance maladie en Chine. Cette étude a pour objectif d'évaluer la disparité de remboursement des frais de prise en charge de la tuberculose parmi les régimes d'assurance maladie indiqués plus haut.
Méthodes: Cette étude a recours à un système de l'Organisation Mondiale de la Santé (OMS) pour analyser les disparités et l'égalité liées aux trois dimensions de l'assurance maladie : la couverture de la population, l'étendue des services couverts et l'étendue dans laquelle les frais sont couverts. Chacune des politiques des régimes d'assurance maladie a été classée et analysée. Une analyse de la base de données des demandes de remboursement relatives à toutes les hospitalisations remboursées de 2010 à 2012 dans trois départements de la ville de Yichang qui contenaient 1506 congés a été menée afin d'identifier les différences en termes de taux de remboursement et de frais restant à la charge des patients parmi les régimes d'assurance maladie.
Résultats: les patients atteints de tuberculose faisaient face à différentes dépenses d'hospitalisation en fonction du régime qui les couvrait (les patients atteints de tuberculose couverts par le NCMS présentent des dépenses d'hospitalisation inférieures à celles des patients couverts par l'URBMI qui eux-mêmes font face à des dépenses d'hospitalisation inférieures à celles des patients couverts par l'UEBMI). Nous avons découvert l'existence d'une importante injustice horizontale en termes de recours aux soins de santé parmi les groupes socio-économiques défavorisés. En termes d'inégalité financière, les patients atteints de tuberculose aux revenus les plus faibles étaient les plus mis à contribution. Le NCMS fournit une protection financière modeste en fonction des revenus. De manière globale, les patients atteints de tuberculose issus de groupes socio-économiques défavorisés étaient les plus vulnérables.
Conclusion: Il existe de vastes disparités en termes de remboursement des soins de prise en charge de la tuberculose parmi les trois régimes d'assurance maladie et cette situation, à son tour, freine le contrôle de la tuberculose. La réduction de l'écart en termes de résultats cliniques entre les trois régimes d'assurance maladie en Chine doit être au cœur des efforts de prise en charge et de contrôle de la tuberculose. L'obtention d'une certaine égalité par l'intermédiaire de politiques intégrées visant à prévenir les discriminations est susceptible d'être efficace.

Translated from English version into French by Eric Ragu, through

Неравенство в возмещении за лечение туберкулезом среди различных схем страхования здоровья: свидетельства из трех уездов в центральном Китае

Яо Пань, Шаньцюань Чэнь, Маньли Чэнь, Пэй Чжан, Цянь Лун, Ли Сян, Генри Лукас

Реферат

Предисловие: Неравенство в охране здоровья является важным вопросом во всем мире. Китайская система базового медицинского обеспечения состоит их трех основных схем страхования, а именно: Базового медицинского страхования городских работополучателей (UEBMI), Базового медицинского страхования городских жителей (URBMI) и Новой кооперативной медицинской схемы (NCMS). В отношении неравенства выплат между схемами страхования в Китае было проведено мало исследований. В настоящем исследовании целью являлась оценка неравенства в возмещении за лечение туберкулеза (ТВ) среди вышеупомянутых схем страхования.

Методы: В данном исследовании для анализа неравенства и равенства в отношении трех аспектов страхования здоровья используются рамки Всемирной организации здравоохранения (ВОЗ) : охват населения, покрываемый диапазон услуг и степень покрытия расходов. Были категорированы и проанализированы все полисы схем страхования здоровья. Был проведен анализ базы данных о претензиях от всех госпитализаций, возмещенных с 2010-го по 2012 г. в трех уездах города Ичан (YC), включавших 1506 случаев выписки из больниц, с целью установления различий в ставках возмещения и собственных расходов среди схем страхования здоровья.

Результаты: Больные туберкулезом несли различные расходы за лечение в стационаре в зависимости от того, какой схемой они были охвачены (пациенты с ТВ, охваченные по NCMS, имеют меньшие расходы за лечение в стационаре, чем пациенты, охваченные по URBMI, которые, в свою очередь, имеют расходы за лечение в стационаре меньшие, чем у охваченных по UEBMI). Нами обнаружено значительное горизонтальное неравенство в практике здравоохранения среди нижних социально-экономических групп. Что касается финансового неравенства, то ТВ-пациенты с меньшими заработками платили больше. NCMS обеспечивает умеренную финансовую защиту на основании дохода. В общем, ТВ-пациенты из нижних социально-экономических групп были наиболее незащищены.

Заключение: Существуют большие различия в возмещении за лечение ТВ среди трех схем страхования здоровья, что, в свою очередь, препятствует контролю за ТВ. Уменьшение разрыва в результатах работы здравоохранения между тремя схемами страхования здоровья в Китае должно быть в фокусе лечения и контроля ТВ. Вероятно, эффективным было бы достижение равенства с помощью интегрированных полисов, избегающих дискриминации.

Translated from English version into Russian by Alexander Somin, through

Disparidad en los reembolsos por atención por tuberculosis en los distintos planes de seguro médico: evidencia de tres condados en China Central.

Yao Pan, Shanquan Chen, Manli Chen, Pei Zhang, Qian Long, Li Xiang, Henry Lucas

Resumen

Antecedentes: La desigualdad en salud es un tema importante a nivel mundial. El sistema de seguridad médica básica en China comprende tres esquemas principales de seguro, a saber el Seguro Médico Básico para Empleados Urbanos (UEBMI), el Seguro Médico Básico para Residentes en Zonas Urbanas (URBMI), y el Nuevo Esquema Médico Cooperativo (NCMS). Se ha llevado a cabo muy poca investigación en cuanto a la disparidad entre los pagos en los distintos esquemas de seguros médicos en China. En este estudio, nuestro objetivo fue el de evaluar la disparidad en los reembolsos por atención por tuberculosis (TB) entre los esquemas de seguro médico antes mencionados.

Métodos: Este estudio usa un marco de la Organización Mundial de la Salud (OMS) para analizar la disparidad y la igualdad en cuanto a tres dimensiones del seguro médico: cobertura de población, el rango de servicios cubiertos, y el alcance de la cobertura de los costos. Se categorizaron y analizaron cada una de las pólizas de los esquemas de seguro médico. Se llevó a cabo un análisis de la base de datos de reclamos de todas las hospitalizaciones reembolsadas entre 2010 y 2012 en tres condados en la ciudad de Yichang (YC), que incluyeron 1.506 altas, con el objetivo de identificar las diferencias en las tasas de reembolsos y los gastos en efectivo entre los esquemas de seguro médico.

Resultados: Los pacientes con tuberculosis tuvieron diversos gastos de hospitalización dependiendo del esquema por el cual estaban cubiertos (los pacientes con TB cubiertos por el NCMS tuvieron menos gastos de hospitalización que aquellos pacientes cubiertos por el URBMI, que tuvieron menos gastos de hospitalización que aquellos cubiertos por el UEBMI). Encontramos una desigualdad horizontal significativa en la utilización de la atención médica entre los grupos socioeconómicos más bajos. En términos de desigualdad financiera, los pacientes con TB que ganaban menos pagaban más. El NCMS ofrece una modesta protección financiera, que se basa en los ingresos. En general, los pacientes con TB de los grupos socioeconómicos más bajos eran los más vulnerables.

Conclusión: Existe una gran disparidad en el reembolso por atención por TB entre los tres esquemas de seguro médico y esto, a su vez, dificulta el control de la TB. El reducir la brecha en los resultados de salud entre los tres esquemas de seguro médico en China debería ser objetivo de la atención y control de la TB. Es muy factible que el logro de la igualdad a través de políticas integradas que eviten la discriminación sea efectivo.

Translated from English version into Spanish by Maria Alejandra Aguada, through

TRANSLATORS
WITHOUT BORDERS
